# Seroprevalence and geographical distribution of human T-lymphotropic virus type 1 among volunteer blood donors in endemic areas of Iran

**DOI:** 10.1186/s12985-017-0693-9

**Published:** 2017-01-30

**Authors:** Gharib Karimi, Maryam Zadsar, Ali Akbar Pourfathollah

**Affiliations:** 1 Blood Transfusion Research Center, High Institute for Research and Education in Transfusion Medicine, Hemmat Exp. Way, Next to the Milad Tower, Tehran, Iran; 2 Department of Immunology, Faculty of Medical Sciences, Tarbiat Modares University, Tehran, Iran

**Keywords:** HTLV-1, Blood donors, Blood donation, Seroepidemiologic studies

## Abstract

**Background:**

Human T-cell lymphotrophic virus type 1 (HTLV-1) has a worldwide distribution and it is endemic in some regions of Iran. One of the most important routes of HTLV-1 transmission is via transfusion of contaminated blood components. The risk of transmission through asymptomatic blood donors, particularly in endemic areas should be considered and appropriately managed. The main objective of this study was to determine the seroprevalence and description the geographic distribution of HTLV-1 among voluntary blood donors in Iran.

**Methods:**

This retrospective study carried out using the data obtained from the main database of the seven blood transfusion centers of Iranian Blood Transfusion Organization between 2009 and 2013. The presence of anti-HTLV-1/2 antibodies were primarily assessed using Enzyme-linked Immunosorbent Assay. The Ab Kit assay, contain antigens for the screening of antibodies to HTLV type 1 and 2. So, it is expressed as HTLV 1/2 assay. Samples that were positive by the western blot confirmatory test were considered as definite positive HTLV-1 or HTLV-2 cases. The main socio-demographic variables were; age, gender, donation history and marital status. Descriptive and analytical statistics were used to summarize the gathered data. The chi-Square Statistical test was used to test the association between groups, P-value of less than 0.05 was considered significant.

**Results:**

A total of 1864489 blood donations were evaluated. There were 1840 confirmed HTLV-1 positive donations (0.098%). None were positive for anti-HTLV-2. The overall HTLV-1 prevalence was 98.7 per 100,000 donations during the 5 year period. Seroprevalence was higher among females, married and older blood donors. The overall seropositivity among first time, regular and lapsed donors was, 0.29% (290/100000), 0.001% (1/100000) and 0.02% (20/100000) respectively. A significant difference was observed between regular and the first time (*p* <0.0001) and also between lapsed and regular blood donors (*p* <0.0001). Most of the HTLV-1 seropositive blood donors (175 per 100,000) were from northeastern regions. We observed a gradual decline in overall HTLV-1 prevalence during the course of the study, the prevalence rate decreased from 0.13% (130/100000) in 2009 to 0.07% (70/100000) in 2013.

**Conclusions:**

The Seroprevalence of HTLV-1 among Iranian blood donors in the regions of our study still is considerable, but there is an obvious declining prevalence over the course of present study. Blood transfusion centers should continually evaluate the residual risk of infection in the country, especially in endemic areas.

## Background

Human T-cell lymphotrophic virus is a retrovirus belonging to the genus deltaretrovirus. Four HTLV related viruses (types 1 to 4) have been detected [1]. HTLV-1 was the first known retrovirus linked to human disease. In addition to Adult T-cell Leukemia and Tropical Spastic Paraparesis, a significant correlation between infection by the virus and diseases such as uveitis, polymyositis, arthritis, autoimmune thyroiditis, Sjogren's syndrome, and infective dermatitis was also reported. However, most HTLV-1 infected people (almost 90%) remain asymptomatic [2, 3]. HTLV-1 has worldwide distribution and infects approximately 5 to 10 million people in the world. However, it is endemic in some regions, such as Japan, Taiwan, Caribbean, Central and South Africa, and some regions of the Middle East [4]. Northeastern Iran is also considered as an endemic area. The first report about the seroepidemiology of HTLV in Iran has been released in 1993 [5]. According to a study has been conducted by Rafatpanah ﻿and his colleagues, the prevalence of HTLV-1 in the general population of northeastern Iran (Mashhad), was estimated to be 2.12% [6]. There are few population-based studies about the endemicity of the virus in other parts of Iran [7, 8]. However, on the basis of seroepidemiologic studies held on blood donors in other parts of the country, HTLV-1 has been reported in the residents of certain areas of Iran [9–16]. The most important routes of HTLV-1 transmission are mother-to-child, especially through breastfeeding, sexual contact and injecting drug users. HTLV-1 can also be transmitted via transfusion of contaminated blood components, liver, kidney and lung transplantation [17]. Following injection of contaminated blood products, the likelihood of HTLV-1 seroconversion is about 40–60% and seroconversion occurs within 60 days in most infected blood recipients [18, 19].

Based on the facts that, the virus has a long duration of asymptomatic phase, high rates of seroconversion after transfusion and considerable prevalence in a certain area, the risk of transmission through asymptomatic blood donors, particularly in high- prevalence areas should be considered and appropriately managed. According to the World Health Organization recommendations, the decision about screening of donations for HTLV-1 infection should be made based on local epidemiological evidences [20]. In northeastern Iran, as an endemic region (including three provinces) laboratory screening for HTLV-1 on blood donations has been started since 1995 [9]. The decision about extending the screening to the blood donations of additional four centers was made by the policy makers in the Iranian Blood Transfusion Organization (IBTO). Currently, HTLV-1 screening test for blood donors is performed regularly just in seven out of 31 provinces.

The main objective of this study was determining the seroprevalence of HTLV-1 antibodies among volunteer blood donors and the associated characteristics in each transfusion center. This study is a comprehensive report on the geographic distribution of HTLV-1 infection among voluntary blood donors in Iran.

## Methods

This retrospective study carried out by using the data obtained from the main database of seven blood transfusion centers of IBTO between the years 2009 and 2013. These centers include Razavi Khorasan, North Khorasan, South Khorasan, Gilan, West Azerbaijan, Ardabil and Alborz which are located in the northeastern, northwestern and north of the central plateau of Iran. A total of 1864489 blood donation records were evaluated. Those who were considered eligible for blood donation were between 18 and 65 years of age and all of them were voluntary donors. All volunteers underwent clinical examination and were interviewed by physicians to record their medical histories and physical findings, especially the history of previous infectious diseases.

As a routine procedure, all selected donors were tested for transfusion transmissible infections, including; HBV, HCV, HIV, Treponema pallidum and HTLV-1/2 at each blood transfusion center. HTLV-1/2 Ab Kit assay, contain a mixture of antigens for the screening of antibodies to HTLV type 1 and 2. So, it is expressed as HTLV 1/2 assay. The presence of anti-HTLV-1/2 antibodies is primarily assessed using commercially available Enzyme-linked Immunosorbent Assay (Adaltis Srl, via Durini, Italy) in a semi-automated system. The presence of antibodies indicates that the blood probably contains viruses. For accurate diagnosis of HTLV-1or HTLV-2 infection, all initially reactive results were further tested by the Western blot technique as a confirmatory test. (MP Biomedical Asia Pacific Pte. Ltd., Singapore). Samples that were positive by western blot confirmatory test were considered as positive samples infected with HTLV-1. In order to consider a test as positive by western blot, two envelope bands (Gd21 and rgp46-I) and at least one core band (p19 with or without p24) had to be positive.

Blood donations with a positive result of HTLV 1& 2 ELISA test had to be discarded and the donor should be excluded from donation permanently, even though without a confirmatory test. Donors who defined as positive by western blot were notified by IBTO centers and referred to a counseling clinic. The main socio-demographic variables were; age, gender, donation type and marital status. Demographic data of seropositive blood donors was fully available and completely extracted, but demographic data of seronegative blood donors were only available in the form of cumulative statistics. Donation histories were differentiated into three types; the first time, lapsed and regular donors. According to IBTO definitions a donor who donated for the first time is a first-time blood donor, a blood donor who has a history of donation, but the interval between two donations is more than 1 year is categorized as lapsed donor and a regular donor is who donated blood more than once during 1 year. In terms of marital status, they were divided into single and married groups.

In order to analyze the data and evaluate the significance of the differences between variables, the annual seroprevalence based on location were calculated per 100,000 blood donations and compared between different geographic areas. In addition, characteristics of seropositive blood donations were studied and reported. Descriptive and analytical statistics were used to summarize the gathered data. The HTLV-1 prevalence (percentage and 95% confidence interval) in each year per 100,000 blood donations was also calculated. The relative risk (RR) was calculated to compare the risks between groups and 95% confidence interval for RRs were also reported. The chi-Square Statistical test was used to test the association between groups and *P*-value of less than 0.05 was considered significant. The reference group was seronegative donations in each variable. This study was approved by the research ethics committee of IBTO.

## Results

**Fig. 1:**
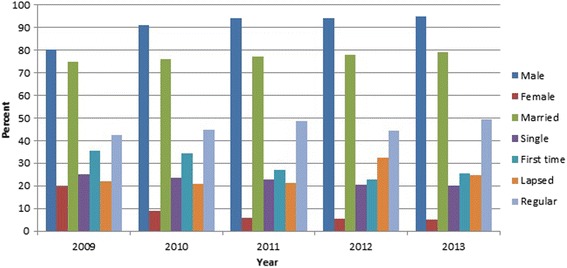
Demographic and donation characteristics among Iranian blood donors during a 5-year period

A total of 1864489 allogeneic blood donations from 2009 through 2013 have been collected at seven blood centers. Geographically, 19.2% of whole country’s donations have taken place in these seven centers. The age distribution of the donors ranged from 18 to 65 years (mean age 34.8 ± 10.4). More than 90% of blood donors were male and 77% were married. Regular donations represented 46% of total donations. The demographic characteristics of blood donors were summarized in Table 1 and Fig. 1.

**Table 1:** Demographic characteristics and history of donation among Iranian blood donors during a 5-year period

Variables	Donors, no. (%)
Gender	
Male	1699811 (91.2%)
Female	164678 (8.8%)
Marital status	
Single	419409 (22.5%)
Married	1438423 (77.1%)
Donation status	
First time	548746 (29.4%)
Regular	857856 (46%)
Elapsed	457887 (24.5%)
Year of donation	
2009	348905
2010	355109
2011	374403
2012	393147
2013	392925
Overall	1864489

There were 1840 donations (0.098%) with confirmed positive HTLV-1 antibody screening tests, all of them were confirmed to be positive for HTLV-1 infection by western blot (95% CI: 0.097–099%). None were positive for anti-HTLV-2. The overall HTLV-1 prevalence was 98.7 per 100,000 donations during the 5 year period (95% CI: 88.6–108 per 100,000). The seroprevalance rate in female and male donors was 300/164678 (0.18%, 95% CI: 0.17–0.19%) and 1540/1699811 (0.09%, 95% CI: 0.09–0.09%), which was shown significant difference (*p* < 0.0001) and RR of two was found in the case of gender seroprevalence (95% CI: 1.77–2.27). The age of seropositive donors ranged between 18 to 65years (mean age 39.4 ± 10.7) which were considerably older (mean age 34.8 ± 10.4) than seronegative donors (*p* < 0.0001). The mean age of female seropositive donor was 42.7 ± 9.9, while the comparative value for the males was 38.8 ± 10.8, shows a significant difference (*p* < 0.0001). The regional HTLV-1 seropositivity rates are shown in Table 2.

**Table 2:** Seroprevalence of HTLV-1 infection among blood donors from seven provinces of Iran (per 100,000)

Location	Donors, no.	Confirmed HTLV-1 +	
		Donors, no.	Prevalence per 100,000 (95% CI)
Alborz	264340	151	57 (50–70)
Ardabil	139613	14	10 (0–21)
Gilan	373227	92	24 (20–30)
North Khorasan	79035	47	59 (40–80)
Razavi Khorasan	628667	1301	207 (200–220)
South Khorasan	72185	24	33 (20–50)
West Azerbaijan	307422	211	68 (60–80)
Total	1864489	1840	98 (97–99)

The seropositivity rate based on donation history, marital status, and gender was given in Table 3. Seropositivity rate among married and single donors were found as 0.11% and 0.05%, respectively. A Significant difference was observed between married and single donors. (*p* < 0.0001), the associated RR was calculated as 1.97 (95%CI: 1.72–2.26). A significant difference was observed between regular and first-time donors (*p* < 0.0001). In addition, the rate of lapsed and regular donors revealed a significant difference (*p* < 0.0001).

**Table 3:** HTLV-1 seropositivity rates based on demographic characteristics and history of donation among Iranian blood donors during a 5-year period

Year	HTLV-1 positive (*n* = 1840) No (%)	Characteristics							
		Mean age	Gender		Marital status		Donation type		
			Male (*n* = 1540) No (%)	Female (*n* = 300) No (%)	Married (*n* = 1603) No (%)	Single (*n* = 237) No (%)	First time (*n* = 1634) No (%)	Elapsed (*n* = 121) No (%)	Regular (*n* = 85) No (%)
2009 2010 2011 2012 2013	475 (0.13%) 419 (011%) 388 (0.1%) 272 (0.069%) 286 (0.07%)	40.20 ± 10.820 39.88 ± 11.133 39.14 ± 10.637 39.28 ± 10.813 38.27 ± 10.184	401 (0.14%) 354 (0.109%) 321 (0.09%) 226 (0.06%) 238 (0.063%)	74 (0.107%) 65 (0.20%) 67 (0.3%) 46 (0.2%) 48 (0.24%)	422 (0.16%) 364 (0.13%) 340 (0.11%) 228 (0.07%) 249 (0.08%)	53 (0.06%) 55 (0.06%) 48 (0.05%) 44 (0.05%) 37 (0.04%)	373 (0.3%) 373 (0.3%) 359 (0.3%) 256 (0.28%) 273 (0.27%)	39 (0.05%) 32 (0.04%) 21 (0.02%) 16 (0.01%) 13 (0.01%)	63 (0.04%) 14 (0.008%) 8 (0.004%) 0 0
*p*-value*			<0.0001		<0.0001		<0.0001		

^*^Chi square test

The distribution of HTLV-1 seropositivity by geographical location showed that the majority (1301/1840 = 70.7%) of HTLV-1 seropositive blood donors were from Razavi Khorasan which is located in northeast of Iran (Table 2). The prevalence rates were significantly different among the seven regions. As expected, the prevalence rates were highest in Mashhad, the center of Razavi Khorasan, followed by Bojnord, the center of North Khorasan Province.

**Fig. 2:**
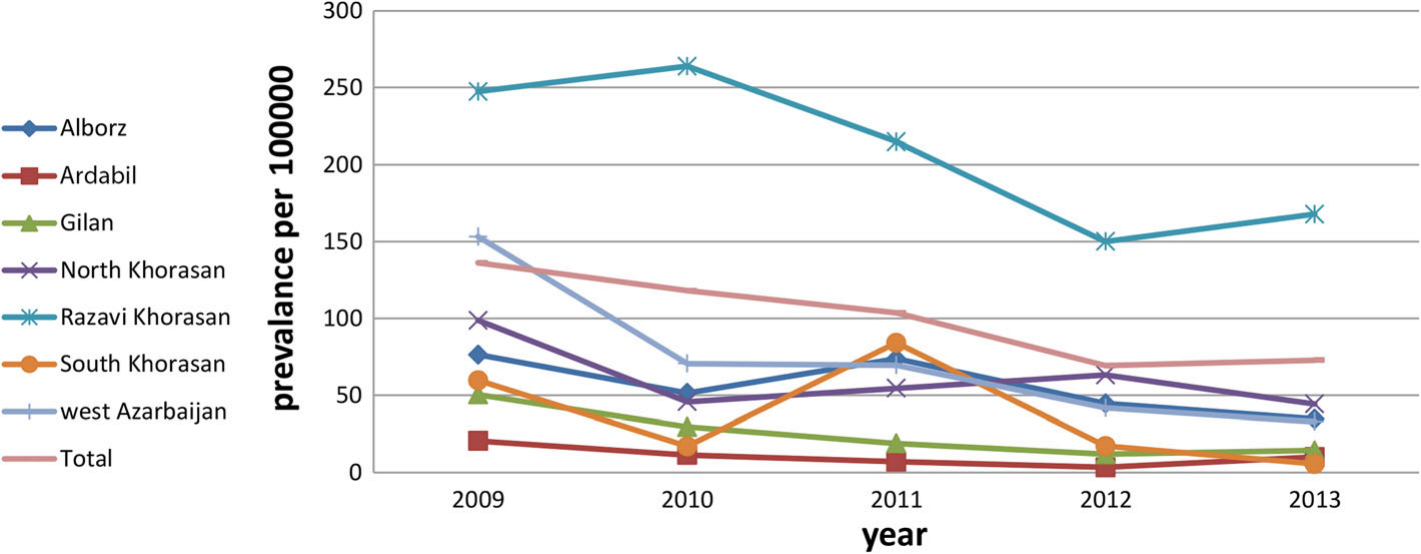
Trend of HTLV-1 seropositivity in seven provinces

We observed a gradual decline in HTLV-1 prevalence during the 5 years, with prevalence decreasing from 0.13% in 2009 to 0.07% in 2013. Figure 2 has shown the trend of HTLV-1 seropositivity in the seven provinces.

## Discussion

**Fig. 3:**
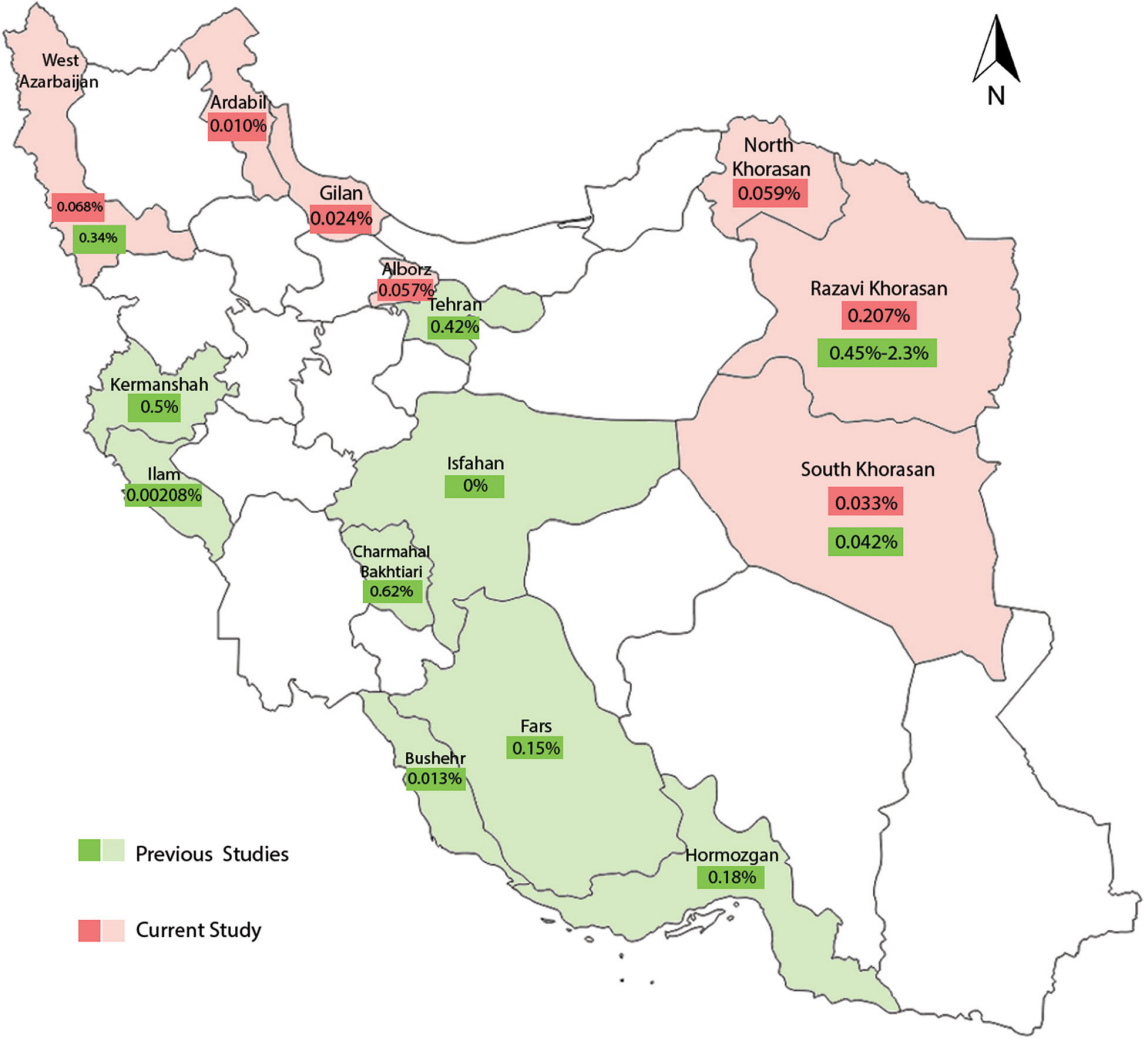
The seroprevalence and geographic distribution of HTLV-1 among blood donors in different provinces

This is a comprehensive study about seroprevalence of HTLV-1 in seven Iranian blood transfusion centers. Through a 5-years survey, we found 1840 confirmed HTLV-1 positive cases among 1864489 donations that indicated a prevalence of 98.7 per 100,000. The HTLV-1 prevalence varies significantly in these seven geographic areas and the highest was seen in donations from Razavi khorasan (207 per 100,000, 95% CI: 200–220). These data confirmed previous findings that khorasan province is an HTLV-1endemic region in Iran [5, 6]. A downward trend in the seroprevalence of HTLV-1 during 2009–2013 was observed. Several surveys with limited sample sizes have been previously conducted to find the prevalence of HTLV-1 in Iranian blood donors [9–16]. These studies have been performed in various regions of the country and have declared the variation in the seroprevalence of HTLV-1 from 0 to 2.3%. The lowest point was reported in the central region of the country (Isfahan) and the highest in the Northeastern regions (Fig. 3). It should be noted that the highest reported frequency is related to a study was done several years ago (1993) in Mashhad. This study reported the prevalence of the HTLV-1 in 2.3% (35/1511) of blood donors [5]. It is conjectured that different factors such as donor selection criterion and laboratory techniques have had an impact on the reporting such a high frequency at the given time. Based on the results of a pilot study conducted in 1995 on blood donors across the country, the prevalence of HTLV-1 in Mashhad was obtained as 1.97% and the prevalence rate in another nine centers was reported between 0.09 and 0.42% [13]. Overall, studies that have been conducted in blood donors in the northeastern regions, have reported a prevalence of 0.77% (219/28487), 0.042% (18/42652) and 0.45% (1054/232648), respectively [14, 15, 17]. As mentioned by these studies, the seroprevalence of HTLV-1 in blood donors over the time has decreased since 1995. In a study held in the general population of Mashhad in 2009, of 1678 analyzed samples, 35 cases were seropositive and the prevalence of infection with HTLV-1 was calculated as high as 2.12%. This study suggests that the prevalence of the virus is still high in Mashhad [6]. The result of our study on blood donors in this city shows the HTLV-1 seroprevalence of 0.28%. This is approximately 7.5 fold less than the general population. This significant difference could be due to the fact that donors are under the process of donor selection and they belong to the low-risk group. Blood donor selection is a rigorous process that is essential to protect the safety of the blood supply. Recruitment of healthy individuals by exploring the risk factors such as the history of HTLV- 1 infection, sexual contacts of individuals with HTLV- 1 infection, family history of HTLV- 1 infection, especially in the mother or maternal grandmother, can be effective to reduce the risk of HTLV- 1 transmission.

HTLV-1 seroprevalence among blood donors in the neighboring countries such as Pakistan, Turkmenistan, and Turkey have been reported at a rate of 0.19% (4/2100), 0.198% (3/1510) and 0.0021% (2/93828), respectively [21–23]. In two studies being conducted on blood donors in Saudi Arabia as a non- endemic country in the region, the HTLV-1 prevalence was 0.006% and 0% [24, 25].

Turkmenistan is Iran's neighbor to the north and northeast. So, Razavi Khorasan and North Khorasan provinces are in direct contact with that country. Reported frequency in Turkmenistan is higher in comparison with the overall frequency in seven provinces and North Khorasan. However, in comparing to Razavi Khorasan, shows the far less frequency. The same situation can be observed in the reported frequency from Pakistan. The result of our study was much higher than those in Turkey and Saudi Arabia.

Among the countries that are known to be endemic, Japan is the first country that performed screening of blood donors for HTLV-1. In a study conducted on 1196321 blood donors in Japan, 3787 donors were confirmed to be positive for anti-HTLV-1 antibody and the overall prevalence of infection was 0.66% in males and 1.02% in females [26]. In endemic areas of Japan the prevalence in blood donors has decreased, but the frequency in the general population was not decreased in the past two decades [27]. The HTLV-1 prevalence in blood donors varies in some other endemic areas such as Brazil 0.04% (387/1038489) [28], Arequipa, Peru 1.2% (35/2732) [29] and Valdivia, Chile 0.24% (15/6237) [30].

HTLV-1 infection prevalence among blood donors in non- endemic areas such as USA (0.05%), France (0.004%), Italy (0.034%), Sweden (0.002%), and Spain (0.001%), is much lower than that in our region [31–35]. It should be noted that most cited studies from other countries were carried out at different times than our study. Several surveys in the endemic and non-endemic regions have shown that, HTLV-1 seroprevalence is significantly associated with the characteristics such as older age, female gender, and lower socioeconomic status [36]. In the present study, a significant relationship between older age and HTLV-1 seroprevalence was observed. This finding is consistent with other studies conducted in the same field and could be due to the cumulative effects of multiple contacts over a lifetime in the endemic areas [5, 6, 15]. Besides, the Prevalence was higher among women, this could be due to the more possibility of transmission from male to female during sexual contacts. As it was shown previously, frequency of infection with the virus in women is twice than men [6, 15, 36]. In terms of marital status, present study revealed that the prevalence of infection in married people was higher than unmarried donors, which might be due to the older age of married people and thus their greater chance of exposure to the infectious agent in addition to higher probability of sexually transmission, although it need further investigations.

In the present study, the prevalence of HTLV-1 infection among first-time blood donors was higher than that of repeated donors. The most probable explanation for this phenomenon is the exclusion effect of screening tests. The first time seropositive donors are not eligible to donate blood again, but it is also possible that a number of first-time donors donate their blood with the motivations such as assessing their health status or due to driving a benefit of blood donation on their health. Also, the prevalence of risky behaviors among first-time donors could be higher than that in regular blood donors due to the process of ongoing blood donor education programs. On the other hand, regular donors are more aware about the blood safety issues due to their longer-lasting relationship with blood transfusion centers [37].

According to the IBTO standard operating procedure, blood donors with HTLV-1 seroreactivity should be permanently deferred from blood donation. This main prevention strategy along with leukocyte reduction procedures (pre-storage method), improvement in blood donor selection procedures, continuous training of employees in the blood transfusion centers, improvement of technical methods, increased awareness among the general population and blood donors about the high-risk behaviors and the modes of virus transmission might have an impact on the reducing the risk of HTLV-1 transmission. It is obvious that the seropositivity has shown a significant variation between geographic areas. The least HTLV-1 seropositivity was seen among blood donors of Ardabil at the rate of 10 per 100,000 donations, so that may be the mandatory screening of this area should be re-evaluated. Currently, apart from the seven provinces, in other parts of the country due to the low prevalence or absence of reported cases, blood donors are not tested for HTLV-1 antibodies﻿. In order to reduce the risk of transmission in areas where routine screening is not performed, it can be suggested that HTLV-1 antibodies testing be performed in donors living in or originating from high-prevalence geographical regions. In addition, asking about the geographic origin of their parents or sexual partner and considering such donors for the screening of HTLV-1 antibodies may be useful to reduce the risk of transmission.

The main strengths of this study are the large sample size, multicentre design, and availability of the computerized database of IBTO. This study has a number of limitations. First, the data for each seronegative donor was not available one by one for all variables (It was recorded as the total number of each category). Second, the data about probable outcomes of positive donors and probable routes of acquisition﻿ of infection﻿ was not available.

## Conclusion

The prevalence of HTLV-1 antibody among Iranian blood donors in the given areas was considerable yet, but apeared to show a descending pattern over 5 years. Besides, the prevalence of HTLV-1 seropositivity is very low in some geographic parts and continuing the screening might be further reevaluated. The most important factors contribute to the declining trend thought to be the strict donor selection process and implication of software system for permanently deferring the seropositive peoples. Although there have been no documented cases of transfusion-transmitted HTLV-1 anywhere in the country yet, blood transfusion centers should continually evaluate the residual risk of infection in donation pool and make appropriate vigilance system, especially in the endemic areas.

## Abbreviations

ATL: Adult T -cell leukemia
HTLV-1: Human T-cell lymphotrophic virus type- I
IBTO: Iranian Blood Transfusion Organization
SOP: Standard operating procedure
TSP: Tropical Spastic Paraparesis
WHO: World Health Organization
